# *De novo* missense variants in FBXO11 alter its protein expression and subcellular localization

**DOI:** 10.1093/hmg/ddab265

**Published:** 2021-09-09

**Authors:** Anne Gregor, Tanja Meerbrei, Thorsten Gerstner, Annick Toutain, Sally Ann Lynch, Karen Stals, Caroline Maxton, Johannes R Lemke, John A Bernat, Hannah M Bombei, Nicola Foulds, David Hunt, Alma Kuechler, Jasmin Beygo, Petra Stöbe, Arjan Bouman, Maria Palomares-Bralo, Fernando Santos-Simarro, Sixto Garcia-Minaur, Marta Pacio-Miguez, Bernt Popp, Georgia Vasileiou, Moritz Hebebrand, André Reis, Sarah Schuhmann, Mandy Krumbiegel, Natasha J Brown, Peter Sparber, Lyusya Melikyan, Liudmila Bessonova, Tatiana Cherevatova, Artem Sharkov, Natalia Shcherbakova, Tabib Dabir, Usha Kini, Eva M C Schwaibold, Tobias B Haack, Marta Bertoli, Sabine Hoffjan, Ruth Falb, Marwan Shinawi, Heinrich Sticht, Christiane Zweier

**Affiliations:** Institute of Human Genetics, Friedrich-Alexander-Universität Erlangen-Nürnberg, Erlangen 91054, Germany; Department of Human Genetics, Inselspital Bern, University of Bern, Bern 3010, Switzerland; Institute of Human Genetics, Friedrich-Alexander-Universität Erlangen-Nürnberg, Erlangen 91054, Germany; Department of Pediatrics, Sørlandet Hospital, Arendal 4838, Norway; Service de Génétique, CHU de Tours, Tours 37044, France; UMR 1253, iBrain, Université de Tours, Inserm, Tours 37044, France; Department of Clinical Genetics, Temple Street Children's Hospital Dublin 1, Dublin D01 YC67, Ireland; Exeter Genomics Laboratory, Royal Devon & Exeter NHS Foundation Trust, Exeter EX2 5DW, UK; Praxis für Kinderneurologie, Hamburg 22767, Germany; Institute of Human Genetics, University of Leipzig Hospitals and Clinics, Leipzig 04103, Germany; Division of Medical Genetics & Genomics, Stead Family Department of Pediatrics, University of Iowa Hospital and Clinics, Iowa City, IA 52242, USA; Division of Medical Genetics & Genomics, Stead Family Department of Pediatrics, University of Iowa Hospital and Clinics, Iowa City, IA 52242, USA; Wessex Clinical Genetics Services, University Hospital Southampton, Southampton SO16 5YA, UK; Wessex Clinical Genetics Services, University Hospital Southampton, Southampton SO16 5YA, UK; Department of Human Genetics and Genomic Medicine, Faculty of Medicine, University of Southampton, Southampton SO16 5YA, UK; Institut für Humangenetik, Universitätsklinikum Essen, Universität Duisburg-Essen, Essen 45147, Germany; Institut für Humangenetik, Universitätsklinikum Essen, Universität Duisburg-Essen, Essen 45147, Germany; Institute of Medical Genetics and Applied Genomics, University of Tübingen, Tübingen 72076, Germany; Department of Clinical Genetics, Erasmus MC University Medical Center Rotterdam, Rotterdam 3015 GD, The Netherlands; Institute of Medical and Molecular Genetics, University Hospital La Paz, Madrid 28046, Spain; Institute of Medical and Molecular Genetics, University Hospital La Paz, Madrid 28046, Spain; Institute of Medical and Molecular Genetics, University Hospital La Paz, Madrid 28046, Spain; Institute of Medical and Molecular Genetics, University Hospital La Paz, Madrid 28046, Spain; Institute of Human Genetics, University of Leipzig Hospitals and Clinics, Leipzig 04103, Germany; Institute of Human Genetics, Friedrich-Alexander-Universität Erlangen-Nürnberg, Erlangen 91054, Germany; Institute of Human Genetics, Friedrich-Alexander-Universität Erlangen-Nürnberg, Erlangen 91054, Germany; Institute of Human Genetics, Friedrich-Alexander-Universität Erlangen-Nürnberg, Erlangen 91054, Germany; Institute of Human Genetics, Friedrich-Alexander-Universität Erlangen-Nürnberg, Erlangen 91054, Germany; Institute of Human Genetics, Friedrich-Alexander-Universität Erlangen-Nürnberg, Erlangen 91054, Germany; Department of Paediatrics, University of Melbourne, Royal Children's Hospital, Melbourne, VIC 3010, Australia; Victorian Clinical Genetics Services, Murdoch Children's Research Institute, Parkville, VIC 3052, Australia; Research Centre for Medical Genetics, Moscow 115522, Russia; Research Centre for Medical Genetics, Moscow 115522, Russia; Research Centre for Medical Genetics, Moscow 115522, Russia; Research Centre for Medical Genetics, Moscow 115522, Russia; Veltischev Research and Clinical Institute for Pediatrics of the Pirogov Russian National Research Medical University, Moscow 125412, Russia; Genomed Ltd., 117997, Moscow, Russia; Veltischev Research and Clinical Institute for Pediatrics of the Pirogov Russian National Research Medical University, Moscow 125412, Russia; Independent Clinical Bioinformatics Laboratory, Moscow 117997, Russia; Department of Genetic Medicine, Belfast City Hospital, Belfast, Northern Ireland BT9 7AB, UK; Oxford Centre for Genomic Medicine, Oxford and Spires Cleft Centre, Oxford OX3 9DU, UK; Institute of Human Genetics, Heidelberg University, Heidelberg 69120, Germany; Institute of Medical Genetics and Applied Genomics, University of Tübingen, Tübingen 72076, Germany; Northern Genetics Service, Newcastle upon Tyne NHS Foundation Trust, Newcastle upon Tyne NE1 3BZ, UK; Department of Human Genetics, Ruhr University, Bochum 44801, Germany; Institute of Medical Genetics and Applied Genomics, University of Tübingen, Tübingen 72076, Germany; Division of Genetics and Genomic Medicine, Department of Pediatrics, Washington University School of Medicine, St. Louis, MO 63110, USA; Institute of Biochemistry, Friedrich-Alexander-Universität Erlangen-Nürnberg, Erlangen 91054, Germany; Institute of Human Genetics, Friedrich-Alexander-Universität Erlangen-Nürnberg, Erlangen 91054, Germany; Department of Human Genetics, Inselspital Bern, University of Bern, Bern 3010, Switzerland

## Abstract

Recently, others and we identified *de novo FBXO11* (F-Box only protein 11) variants as causative for a variable neurodevelopmental disorder (NDD). We now assembled clinical and mutational information on 23 additional individuals. The phenotypic spectrum remains highly variable, with developmental delay and/or intellectual disability as the core feature and behavioral anomalies, hypotonia and various facial dysmorphism as frequent aspects. The mutational spectrum includes intragenic deletions, likely gene disrupting and missense variants distributed across the protein. To further characterize the functional consequences of *FBXO11* missense variants, we analyzed their effects on protein expression and localization by overexpression of 17 different mutant constructs in HEK293 and HeLa cells. We found that the majority of missense variants resulted in subcellular mislocalization and/or reduced FBXO11 protein expression levels. For instance, variants located in the nuclear localization signal and the N-terminal F-Box domain lead to altered subcellular localization with exclusion from the nucleus or the formation of cytoplasmic aggregates and to reduced protein levels in western blot. In contrast, variants localized in the C-terminal Zn-finger UBR domain lead to an accumulation in the cytoplasm without alteration of protein levels. Together with the mutational data, our functional results suggest that most missense variants likely lead to a loss of the original FBXO11 function and thereby highlight haploinsufficiency as the most likely disease mechanism for *FBXO11*-associated NDDs.

## Introduction

Recently, *de novo* variants in the F-Box protein encoding *FBXO11* gene have been described as causative for a variable neurodevelopmental disorder [MIM #618 089, intellectual developmental disorder with dysmorphic facies and behavioral anomalies (IDDFBA); (1–3)]. Very recently, the first familial case of IDDFBA has also been reported (4). To date, 51 individuals from 49 independent families with pathogenic or likely pathogenic *FBXO11* variants have been described (1–6). Affected individuals show variable degrees of cognitive impairment ranging from normal IQ with developmental delay (DD) to severe intellectual disability (ID). Behavioral anomalies are common. Seizures and brain abnormalities are observed in a fraction of individuals (1–4). Growth parameters are highly variable with short stature and microcephaly being more common than tall stature and macrocephaly. Other frequently reported features include skeletal abnormalities, recurrent infections and vision anomalies. Though facial dysmorphism is observed in the majority of individuals, no recognizable facial gestalt could be delineated (2,3). The mutational spectrum encompasses large multi-gene and intragenic deletions, likely gene-disrupting (LGD) variants, single amino acid deletions and missense variants. The latter are distributed across the protein and mostly localize to any of the protein domains. No obvious genotype–phenotype correlations could be established (2,3). It has therefore been postulated that haploinsufficiency might be the most likely disease mechanism for all described *FBXO11* variants, but gain-of function or dominant negative effects could not be excluded (2,3).

FBXO11 belongs to the F-Box protein family, which contains over 60 members that all carry an F-Box domain in addition to various other protein domains (7,8). FBXO11 contains several functional domains, including the eponymous F-Box domain, important for interaction with S-phase kinase-associated protein 1 (SKP1) and subsequently other SCF complex components (9). It also contains three carbohydrate binding/sugar hydrolysis (CASH) domains, which are important for substrate recognition (7,10) and a Zinc-finger UBR (Zf-UBR) domain, which interacts with N-terminal degradation signals in substrate proteins and is a characteristic feature of E3 ubiquitin ligases (11). FBXO11 constitutes a subunit of an E3-ubiquitin ligase complex, the SCF complex (SKP1-Cullin-F-Box), which additionally includes SKP1 and Cullin 1 [CUL1 (7)]. Within the complex, FBXO11 is important for recognition of substrates resulting in their ubiquitination and subsequent degradation. It is also thought to play a role in maintenance of genome stability (12). Its function has mainly been studied in the context of different malignancies such as B-cell lymphoma (10), myelodysplastic syndrome (13), hepatocellular carcinoma (14), gastric cancer (15) and glioblastoma (16). The functional role of FBXO11 in neurodevelopmental disorders (NDDs) and neurodevelopment in general remains elusive. Germline variants in several other F-Box proteins have also been linked to NDDs inherited either in autosomal recessive (17–22) or in autosomal dominant fashion (23–26). Interestingly, defects in genes involved in protein ubiquitination and proteasomal degradation have recently emerged as a common theme among NDDs [e.g. (27–35)].

We now further delineate the clinical and mutational spectrum of a *FBXO11*-associated NDD by adding 23 additional cases with *de novo FBXO11* variants identified through either chromosomal microarray testing or exome sequencing. Furthermore, we characterize the functional effects of several published and the majority of the novel *FBXO11* missense variants. Our results show impaired expression or subcellular localization for the majority of the missense variants, supporting the hypothesis that *FBXO11* deficiency is caused by loss of function.

## Results

### Further delineation of the clinical spectrum

**Figure 1:**
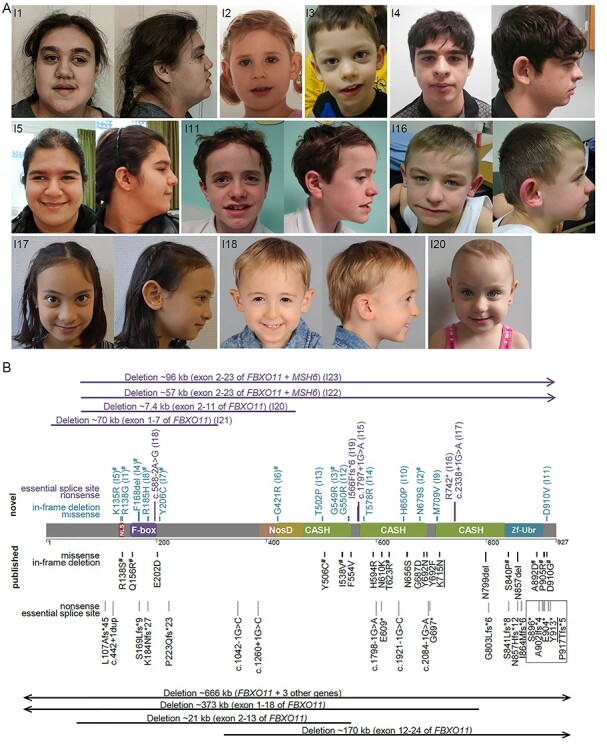
Overview of the clinical and mutational spectrum in individuals with *FBXO11* aberrations. (**A**) Clinical images of affected individuals. (**B**) Schematic drawing of FBXO11 (NM_001190274.1, NP_001177203.1) with annotation of novel [top, blue (missense variants) and purple (LGD variants)] and previously published (bottom, black) aberrations. Domains are color-coded according to InterPro (66). The spectrum encompasses large multi-gene and intragenic deletions, LGD variants, single amino acid deletions and missense variants. Most variants are unique, recurrent variants are underlined. All variants included in the functional assays are marked with the symbol of hash. LGD variants predicted to escape nonsense mediated decay are marked with a black box. Single letter amino acid codes were used due to space constraints.

All 23 individuals with *FBXO11* aberrations presented with DD and/or ID. Cognitive impairment was variable, ranging from normal IQ with learning difficulties and attention deficits in two individuals to severe ID in three individuals. Developmental milestones were delayed in many cases, with age of walking ranging from 8 months (normal) to no walking ability at age 10 years and age of first words ranging from normal development with bilingual upbringing to no meaningful verbal communication at age 10 years. Behavioral anomalies were commonly observed among individuals and included attention deficits, anxiety and autism. Seizures were reported in nine individuals, and two additional individuals showed abnormal EEG but no seizures. Body measurements were also variable, and in the normal range for the majority of cases, with short stature observed in three individuals. Head circumference was also in the normal range for the majority of cases, with three individuals reported as microcephalic. Increased body weight and/or hyperphagia were reported in four individuals. Non-specific facial dysmorphism were reported in most individuals (Fig. 1A). Recurrent infections and/or otitis media were reported in 9/20 individuals. Unspecific MRI abnormalities were present in 9/20 individuals and included white matter abnormalities, thin corpus callosum and arachnoidal cyst. Abnormalities of hand and feet were reported in 16/22 individuals and included polysyndactyly, severe pronation with hindfoot malformation, shortened metacarpals and nail hypoplasia. Skeletal anomalies such as rhizomelic shortening of arms and legs, scoliosis, kyphosis, enthesitis-related juvenile idiopathic arthritis and delayed bone age were reported in 10/22 individuals. Vision anomalies were found in 10/22 individuals and included strabismus, myopia and hypermetropia. Hearing impairments were reported in two cases. Other abnormalities included bowel lymphangiectasia, hyper-IgD syndrome, chronic increase of blood sedimentation rate without inflammatory signs and feeding difficulties in one patient each. A summary of all clinical information can be found in Table 1 and detailed clinical information for each individual can be found in Supplementary Material, Table S1.

**Table 1:** Overview of phenotypes in novel and published cases and phenotypes

	Novel^a^ (%)	Published^b^ (%) (1–5)	Total %	Total (%) (*P*-value versus missense^*^)			
Variant type	All	All	All	Missense/single aa deletion	LGD all	LGD w NMD	LGD w/o NMD
*n*	22	49	71	*n* = 34	*n* = 37	*n* = 30	*n* = 7
ID/DD	22/22 (100%)	49/49 (100%)	100%	34/34 (100%)	37/37 (100%)	30/30 (100%)	7/7 (100%)
Mild	15/22 (68%)	23/49 (47%)	54%	18/34 (53%)	20/37 (54%)	18/30 (60%)	2/7 (29%)
Moderate	5/22 (23%)	18/49 (37%)	32%	10/34 (29%)	13/37 (35%)	10/30 (33%)	3/7 (42%)
Severe	2/22 (9%)	8/49 (16%)	14%	6/34 (18%)	4/37 (11%)	2/30 (7%)	2/7 (29%)
Seizures/abnormal EEG	10/22 (45%)	10/47 (21%)	29%	13/33 (39%)	7/36 (19%)	6/30 (20%)	1/6 (17%)
Hypotonia	14/20 (70%)	30/43 (70%)	70%	22/31 (71%)	22/32 (69%)	16/26 (62%)	6/6 (100%)
Facial dysmorphism	21/22 (95%)	44/46 (96%)	96%	30/33 (91%)	35/35 (100%)	29/29 (100%)	6/6 (100%)
Microcephaly	3/20 (15%)	12/48 (25%)	22%	4/32 (13%)	11/36 (31%)	8/29 (28%)	3/7 (43%)
Macrocephaly	0/20 (0%)	3/48 (6%)	4%	2/32 (6%)	1/36 (3%)	1/29 (3%)	0/7 (0%)
Overweight/hyperphagia	4/22 (18%)	19/49 (39%)	32%	6/34 (18%)	**17/37 (46%) (0.013)**	**15/30 (50%) (0.008)**	2/7 (29%) (ns)
Short stature	3/21 (14%)	11/48 (23%)	20%	9/33 (27%)	5/36 (14%) (ns)	**2/29 (7%) (0.048)**	3/7 (43%) (ns)
Tall stature	0/21 (0%)	4/48 (8%)	6%	2/33 (6%)	2/36 (6%)	2/29 (7%)	0/7 (0%)
Behavioral anomalies	12/20 (60%)	36/48 (75%)	71%	22/33 (67%)	26/35 (75%)	21/28 (75%)	5/7 (71%)
Vision impairment	10/21 (48%)	22/45 (49%)	48%	18/31 (58%)	14/35 (40%)	9/29 (31%)	5/6 (83%)
Recurrent infections	9/19 (47%)	9/23 (39%)	43%	10/20 (50%)	8/22 (36%)	6/19 (32%)	2/3 (67%)
MRI abnormalities	8/19 (42%)	14/32 (44%)	43%	15/29 (52%)	7/22 (32%)	6/17 (35%)	1/5 (20%)

*Notes:* For individual clinical information see Supplementary Material, Tables S1 and S3, nominally significant *P*-values are highlighted in bold, all *P*-values not indicated were not significant; LGD: likely gene-disrupting variants including frameshift mutations, nonsense mutations, splice site variants and intragenic deletions; NMD: nonsense-mediated decay; ns: not significant.

a Individual I8 from the current study was excluded from phenotypic summary due to dual diagnosis.

b For the familial cases from Lee et al. 2020 only individual 1 was included here.

^^*^^Significance was calculated using a chi-square test by comparison of number cases with missense variants compared to numbers with different LGD categories (all LGDs, LGD with predicted NMD and LGD without predicted NMD).

### Further delineation of the mutational spectrum

In 23 individuals, we identified four deletions, five LGD variants, a single amino acid deletion and 13 missense variants affecting *FBXO11* (Fig. 1B). All of these variants were shown to have occurred *de novo*, and for the majority paternity was confirmed through trio exome analysis. Two deletions affected *FBXO11* only partially and deleted exons 1–7 (I21) or 2–11 (I20). Both deletions are predicted to lead to loss of the affected allele through either loss of the start codon of all isoforms (I21), or an out-of-frame deletion of more than a third of the protein including the F-Box domain (I20). The other two deletions affected exons 2–23 of *FBXO11* as well as the neighboring gene *MSH6* (I22 and I23). LGD variants included three splice variants affecting canonical splice sites. Two of these variants (c.588-2A > G and c.1797 + 1G > A) are predicted to lead to out-of-frame exclusion of exons 5 and 14, respectively. The third splice variant (c.2338 + 1G > A) is predicted to lead to the exclusion of exon 19, which is predicted to result in an in-frame deletion of 37 amino acids affecting more than one fourth of the third CASH domain. Lack of patient-derived material precluded further testing of splice variants. The identified nonsense p.(Arg742^*^) and frameshifting p.(Ile566Phefs^*^6) variants are predicted to lead to nonsense-mediated mRNA decay (NMD). All 13 missense variants affected highly conserved amino acid residues (Supplementary Material, Fig. S1), were absent from public databases such as gnomAD (36), and most were predicted to be damaging by several *in-silico* prediction programs, but not to affect splicing [(37); Supplementary Material, Table S2). The missense variants are distributed across the protein and reside in different domains. Variants localized to the putative nuclear localization signal (NLS) as annotated by cNLS mapper [(38); p.(Lys135Arg) and p.(Arg138Gly)], in or around the F-Box domain [p.(Arg185His) and p.(Tyr206Cys)], in the CASH domains 1 [p.(Gly421Arg), p.(Thr502Pro), p.(Gly549Arg) and p.(Gly550Arg)], 2 [p.(Thr578Arg), p.(His650Pro) and p.(Asn679Ser)] and 3 [p.(Met709Val)], or in the C-terminus of the protein [p.(Asp910Val)]. The single amino acid deletion affected a highly conserved position in the F-Box domain p.(Phe168del). All observed variants were unique and affected distinct amino acid positions, except for p.(Arg138Gly) and p.(Asp910Val), where a different amino acid exchange has been reported at the same position (2). According to the American College of Medical Genetics and Genomics (ACMG) guidelines (39), all variants identified here are considered likely pathogenic or pathogenic (Supplementary Material, Tables S1 and S3).

### Assessment of genotype–phenotype correlations

We considered the group of individuals described here and summarized from previous reports large enough [*n* = 71, 49 independent published cases (1–6) + 22 novel cases] to attempt characterizing genotype–phenotype correlations. For those correlations, we excluded individual I8 who additionally has Koolen–de Vries syndrome (MIM #610 443) due to a pathogenic variant in *KANSL1*, confounding the clinical picture. Summarized clinical data of all cases can be found in Supplementary Material, Table S3. Overweight/hyperphagia were more common in individuals carrying LGD variants compared to carriers of missense variants with a nominally significant *P*-value [Table 1, chi-square test, 46% versus 18% (*P* = 0.013)]. When comparing missense variants with only LGD variants predicted to lead to NMD (NMD–LGD), we furthermore found that affected individuals carrying missense variants are significantly more often of short stature compared with NMD-LGD carriers [27% versus 7% (*P* = 0.048)]. Regarding short stature, carriers of LGDs that are predicted to escape NMD (no-NMD–LGD) are phenotypically more similar to missense variant carriers than to NMD–LGD carriers, and regarding overweight the differences between LGD and missense variant carriers seem to be driven mainly by the NMD–LGD variant carriers. Other clinical features such as the presence of seizures, behavioral anomalies, hypotonia, facial dysmorphism, MRI abnormalities, vision abnormalities or recurrent infections were equally common across groups with different mutation types. One factor that may influence the ability to draw any genotype–phenotype correlations is the presence of additional *de novo* variants of uncertain significance in other genes that was found in six of the previously published cases (2,3). It must be noted that none of the observed differences remain statistically significant when correcting for multiple testing. Thus, even larger clinical cohorts will be needed to define potential differences. In summary, while we can observe subtle phenotypic differences between carriers of either missense or LGD variants, no distinct phenotypic groups could be established.

### *FBXO11* missense variants are predicted to destabilize the protein

**Figure 2:**
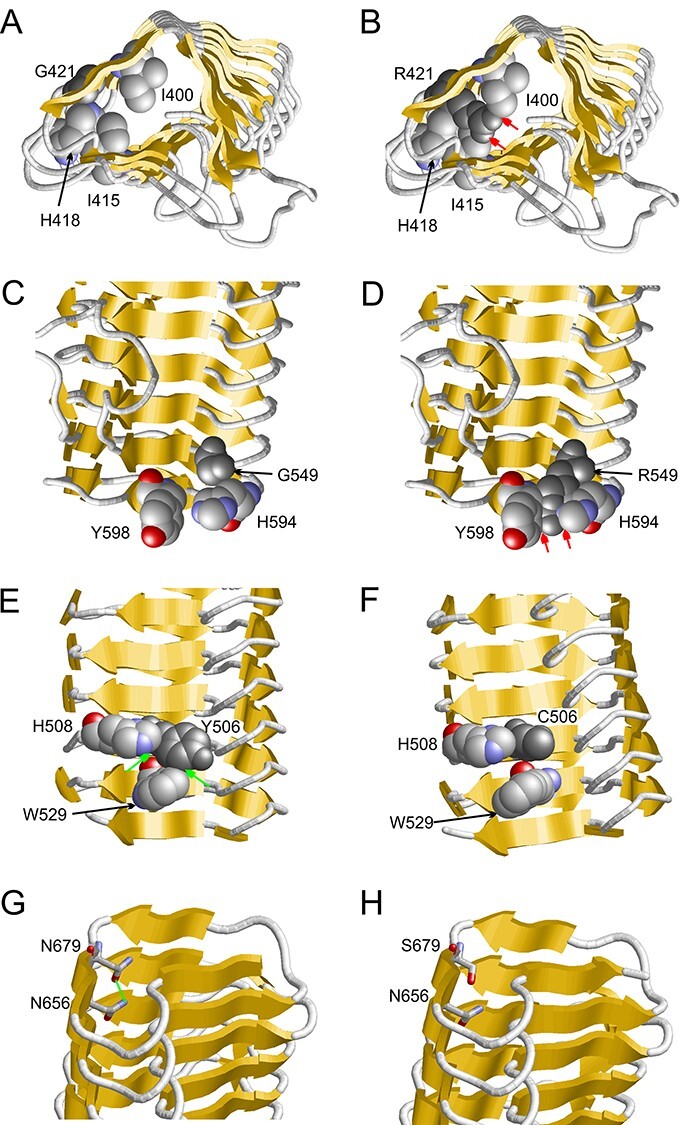
FBXO11 variants affect stability of the protein. Results of mutational modelling for three novel [p.(Gly421 Arg), p.(Gly549His) and p.(Asn679Ser)] and one previously published missense variants [p.(Tyr506Cys; (3) ]are shown. (**A**) Vicinity of G421 (dark grey) within the β-helix (β-strands are indicated as yellow arrows and key residues are shown in space-filled presentation). (**B**) The R421 sidechain (dark grey) points towards the interior of the β-helix and forms steric clashes (red arrows) with the sidechains of I400, I415 and H418. (**C**) Vicinity of G549 within the β-helix. (**D**) The bulkier R549 sidechain forms steric clashes (red arrows) with the sidechains of H594 and Y598. (**E**) Y506 forms hydrophobic interactions (green arrows) with H508 and W529. (**F**) These interactions cannot be formed by the shorter cysteine sidechain in the Y506C variant. (**G**) N679 forms a sidechain hydrogen bond (green line) to N656. (**H**) This interaction cannot be formed by the shorter serine sidechain in the N679S variant.

To further investigate possible effects of missense variants on protein stability and function we first performed *in-silico* mutational modelling for six novel variants and one previously published variant [p.(Tyr506Cys); (3)] in domains where structural information is available. The CASH domains of FBXO11 exhibit an elongated structure that consists of right-handed β-helices (40). All variants in this domain have an unfavorable effect on the protein structure either by causing a loss of stabilizing interactions or by inducing steric problems [‘clashes’; (Fig. 2)]. In the Gly421Arg and Gly549Arg variants, tiny glycines (Fig. 2A and C) are replaced by much bulkier arginines, which results in steric clashes (Fig. 2B and D) that potentially destabilize the protein structure. In case of the Tyr506Cys and Asn679Ser variants the wildtype residues form stabilizing hydrophobic (Y506; Fig. 2E) or polar (N679; Fig. 2G) interactions with adjacent residues. In both variants, the wildtype residue is replaced by an amino acid with a shorter sidechain, which could result in a loss of these stabilizing interactions (Fig. 2F and H). In the His650Pro variant, the proline is sterically not tolerated and therefore expected to destabilize the domain structure (not shown). Modelling of the variants located in the F-box domain suggests that they also disturb the domain structure, either by a loss of polar interactions (Arg185His) or by the loss of Phe168 that is central part of the hydrophobic core (Phe168del).

### SCF complex formation is not affected by *FBXO11* missense variants

FBXO11 is part of the SCF ubiquitin ligase complex (7). To test whether any of the missense variants affect formation of the basic SCF complex, we performed co-immunoprecipitation experiments following transient overexpression of Myc-tagged wildtype or mutant FBXO11 for nine of the previously reported and one novel missense variants (see Table 2). All tested missense variants were still able to co-immunoprecipitate CUL1, another subunit of the SCF complex, at similar levels (Supplementary Material, Fig. S2), suggesting that SCF-complex formation is not impaired by the tested missense variants.

**Table 2:** Overview of functional testing results of *FBXO11* missense variants

Variant^a^	Domain^b^	Modelling	Complex formation	Localization	Expression level		Variant reported
WT				N + C	100		
K135R (K51R)	NLS	Np	Nd	C	47 ± 14	0.03	This study
R138G (R54G)	NLS	Np	Normal	C	60 ± 10	0.02	This study
R138S (R54S)	NLS	Np	Normal	C	53 ± 14	0.03	(2)
Q156R (Q72R)	F-Box	Np	Normal	Granula	49 ± 13	0.03	(2)
F168del (F84del)	F-Box	Loss of stabilizing interaction	Nd	Granula	69 ± 7	0.02	This study
R185H (R101H)	F-Box	Loss of stabilizing interaction	Nd	Granula	55 ± 14	0.04	This study
Y206C (Y122C)		Np	Nd	N + C	43 ± 8	0.004	This study
G421R (G337R)	CASH 1	Steric clashes	Nd	N + C	55 ± 9	0.009	This study
T502P (T418P)	CASH 1	Nd	Nd	Nd	Nd	Nd	This study
Y506C (Y422C)	CASH 1	Loss of stabilizing interaction	Normal	Granula	59 ± 12	0.03	(3)
I538V (I454V)	CASH 1	Loss of stabilizing interaction^c^	Normal	Granula	98 ± 6	0.8	(2)
G549R (G465R)	CASH 1	Steric clashes	Nd	Granula	47 ± 10	0.01	This study
G550R (G466R)	CASH 1	Nd	Nd	Nd	Nd	Nd	This study
T578R (T494R)	CASH 2	Nd	Nd	Nd	Nd	Nd	This study
T623R (T539R)	CASH 2	Electrostatic repulsion^c^	Normal	Granula	106 ± 12	0.7	(2)
H650P (H566P)	CASH2	Destabilization of domain structure	Nd	Nd	Nd	Nd	This study
N679S (N595S)	CASH 2	Loss of stabilizing interaction	Nd	N + C	57 ± 6	0.005	This study
M709V (M625V)	CASH 3	Np	Nd	Nd	Nd	Nd	This study
S840P (S756P)	Zf-UBR	Steric clashes^c^	Normal	Granula, coloc. With SCF	116 ± 27	0.6	(2)
A892D (A808D)	Zf-UBR	Conformational change^c^	Normal	Granula	120 ± 21	0.4	(2)
P905R (P821R)		Np	Normal	N + C	95 ± 30	0.9	(2)
D910G (D826G)		Np	Normal	Granula	118 ± 21	0.4	(2)
D910V (D826V)		Np	Nd	Nd	Nd	Nd	This study

Nd: not determined, Np: not possible, N: nuclear, C: cytoplasmic.

a Annotation according to the longest isoform NP_001177203.1 (and shorter cloned isoform NP_079409.3).

b Domain structure according to Interpro.

c Reported in Gregor *et al.* (2).

### *FBXO11* missense variants affect subcellular localization

**Figure 3:**
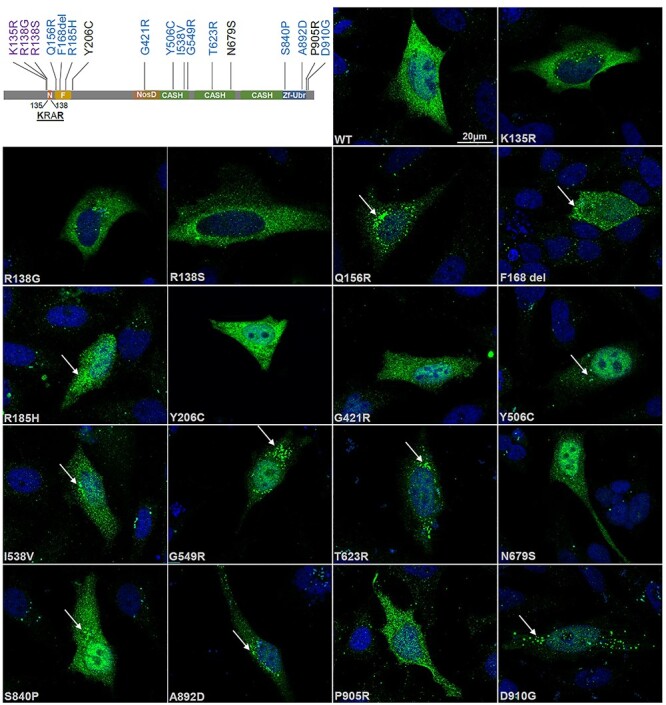
FBXO11 variants affect subcellular localization. Schematic drawing of FBXO11 on top left indicates variants with altered subcellular localization in different colors (nuclear exclusion: purple, cytoplasmic aggregates: blue). For immunofluorescence analysis HeLa cells were transiently transfected with 400 ng per 12 well of wildtype or mutant Myc-FBXO11. Cells were fixed 48 h post transfection and stained with an anti-Myc antibody. Images were taken on a Axioimager Z2 with Apotome. Scale bar 20 μM. Arrows point to subcellular aggregates present in several mutants.

FBXO11 localizes to the cytoplasm and the nucleus in HeLa cells (Fig. 3). To investigate whether missense variants might lead to subcellular mislocalization we tested the expression of Myc-tagged FBXO11 constructs each carrying 1 of 17 missense variants either described here or published previously [Table 2; (2)] and quantified the observed phenotypes. Missense variants p.(Lys135Arg), p.(Arg138Ser) and p.(Arg138Gly), all located in the putative NLS sequence, abrogated subcellular localization and led to exclusion from the nucleus in all transfected cells (Fig. 3). Furthermore, many of the missense variants located in the F-Box domain [p.(Gln156Arg) and p.(Arg185His)], CASH domains [p.(Tyr506Cys), p.(Ile538Val), p.(Gly549Arg) and p.(Thr623Arg)] and Zf-UBR domain [p.(Ser840Pro) and p.(Ala892Asp)] or at the C-terminus of the protein adjacent to the Zf-UBR domain [p.(Asp910Gly)] led to the formation of cytoplasmic aggregates in 50–90% of transfected cells (Fig. 3 and Table 2, quantified in Supplementary Material, Fig. S3A). Similar aggregates were present in only 10% of wildtype cells. To rule out that the observed aggregates are purely an overexpression artifact, we confirmed the presence of the aggregation phenotype for some of the variants also when transfecting lower doses of *FBXO11* containing plasmid (200 ng per 12 well versus 400 ng per 12 well; Supplementary Material, Fig. S3B and C). We furthermore also confirmed the formation of cytoplasmic aggregates albeit at a lower frequency of 30–50% of cells for some variants in another cell type, HEK293 cells (Supplementary Material, Fig. S4). To investigate the nature of these aggregates, we picked several of the aggregate forming variants from the different domains [F-Box: p.(Gln156Arg), CASH 1: p.(Gly549Arg) and CASH 2: p.(Thr623Arg)] and performed co-staining with markers for different subcellular organelles, namely the endoplasmic reticulum (ER), the Golgi apparatus, and early or late endosomes/lysosomes. The aggregates did not co-localize with any of the markers, suggesting that they are not associated with any organelle (Supplementary Material, Fig. S5). We next tested whether these aggregates contain other components of the SCF complex by co-staining with antibodies against CUL1 and SKP1. Although most variants tested did not show evidence of co-localization with the SCF complex (Supplementary Material, Fig. S6), we found that aggregates of variant p.(Ser840Pro) partially co-localize with CUL1 in the cytosol, suggesting that some, but not all of those aggregates may be SCF complexes (Supplementary Material, Fig. S6). We finally performed co-staining with an FK2-antibody, which stains ubiquitinated proteins. However, we were also not able to find any evidence of co-localization of aggregates with the FK2 signal (Supplementary Material, Fig. S7), leaving the nature of the observed aggregates unclear for the majority of variants.

### Protein expression is reduced for many *FBXO11* missense variants

**Figure 4:**
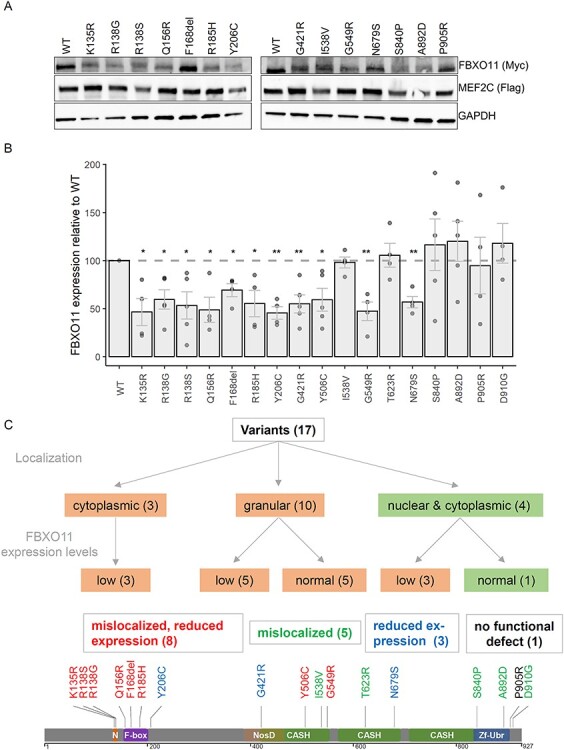
FBXO11 variants affect protein expression levels. (**A**) Representative image of western blots used for quantification of FBXO11 expression levels is shown. (**B**) Quantification of FBXO11 protein levels from western blotting. Value of wildtype FBXO11 was set to 100. Experiments were repeated at least 4 times. Individual values are shown as dots with mean values shown as bars with SEM. *P*-values were calculated using a one sample t-test with the hypothetical mean set to 100 and a significance threshold of < 0.05 (^*^ < 0.05, ^*^^*^ < 0.01). (**C**) Schematic overview of different functional consequences observed for tested missense variants with schematic drawing of protein with mutations color-coded according to results of functional assays (red: mislocalization and reduced expression, green: mislocalization, blue: reduced protein expression, black: no functional defect observed).

We also investigated whether *FBXO11* missense variants affect protein expression. We therefore performed western blotting analysis after overexpression of Myc-tagged wildtype or mutant FBXO11. To control for transfection differences we co-transfected a FLAG-tagged control protein (MEF2C), which was used for quantification of blots. We found that missense variants located in the NLS and the F-Box domain all resulted in reduced expression levels of FBXO11 (Fig. 4). For missense variants located in one of the three CASH domains, expression was more variable and reduced in four mutant constructs [p.(Gly421Arg), p.(Tyr506Cys), p.(Gly549Arg) and p.(Asn679Ser)], whereas it remained unchanged for two others [p.(Ile538Val) and p.(Thr623Arg)]. Variants affecting the Zf-UBR domain or the C-terminus of the protein did not seem to alter FBXO11 expression.

### Integration of functional assay data

*FBXO11* missense variants can lead to a spectrum of functional effects (Fig. 4C), ranging from abnormal localization combined with reduced protein expression for eight variants, abnormal localization with normal protein levels for five variants or reduced protein expression with normal localization for three variants. Only one variant did not show any functional defects in the assays tested here [p.(Pro905Arg)]. N-terminal variants localized in the NLS or the F-Box domain all affect localization and stability of FBXO11. C-terminal variants located in the Zf-UBR domain or the very C-terminus are likely to affect localization, but not protein expression levels. For the variants in the CASH domains, no specific pattern could be observed with all variants affecting either localization, expression levels or both.

## Discussion

With 23 novel cases reported in this study, a total of 74 individuals from 72 independent families with pathogenic or likely pathogenic *FBXO11* aberrations according to the ACMG guidelines have been described to date (1–5). The clinical spectrum among all cases remains highly variable with DD and/or ID of varying severity as the only feature present in all individuals. Clinical variability is also reflected by the fact that the first familial cases with a LGD variant in *FBXO11* and a mild phenotype have been described recently (4). Most pathogenic variants in *FBXO11*, however, have occurred *de novo*. The most frequent additional clinical features include behavioral anomalies, hypotonia, vision impairment and variable non-specific facial dysmorphism alongside with various other anomalies. Recurrent infections and/or otitis media have been reported in several cases, which is notable as also a heterozygous *Fbxo11* mouse model showed increased susceptibility to chronic otitis media (41), which may point to a role of FBXO11 in immune response. Cleft lip/palate has been reported in five individuals, but does not seem to be a common feature in *FBXO11*-associated NDDs, though reported in two homozygous *Fbxo11* mouse models (41). Of note, the deletions in individuals I22 and I23 as well as three published cases (2,3,6) also included the neighboring *MSH6* gene, loss-of-function variants in which are responsible for Lynch syndrome (MIM #120 435). These individuals may therefore have an increased risk of colon cancer and other types of tumors (42,43). Very recently, a potential role for FBXO11 in a diffuse large B-cell lymphoma was discussed in an individual with DD and a deletion of *FBXO11* and *MSH6* (6). Larger cohorts and longitudinal monitoring are necessary to elucidate whether in addition to somatic *FBXO11* variants also germline variants may predispose to various malignancies. It is noteworthy that for several NDD genes, in which somatic variants have been associated with various malignancies, no increased tumor risk has been observed in NDD cases so far, for example *ARID1B*, germline variants in which cause Coffin-Siris syndrome [MIM#135 900; (44)].

In addition to the clinical variability, *FBXO11*-associated NDDs also show variability in the mutational spectrum, which ranges from different types of intragenic deletions and LGD variants to missense variants and single amino acid deletions. No mutational hotspots or preferred mutation types seem to be present, which is in line with the intolerance of *FBXO11* to loss-of-function variants [pLI = 1; (45)] and the findings that missense variants in haploinsufficient genes tend to not cluster in specific protein regions (46). Also the C-terminal clustering of missense and likely-gene disrupting variants observed in our initial report (2) is not confirmed in this larger cohort. It is notable, however, that seven of the LGD variants are located at the very C-terminus of *FBXO11* in the last exon and are therefore predicted to escape nonsense-mediated decay (47). This may result in a shorter stable protein that lacks part of the Zf-UBR domain or in a shorter protein with abnormal folding, abnormal stability or reduced half-life. Lack of patient-derived material, however, precluded functional testing. Through investigating genotype–phenotype correlations, we found that obesity may be more common in individuals carrying LGD variants compared with missense variant carriers. In addition, short stature may be more common in individuals carrying missense variants compared with carriers of LGD variants that are predicted to lead to NMD. Those potential differences, however, are subtle and insufficient to establish distinct phenotypic groups related to particular variants. Therefore, the lack of clear phenotype separation based on different variant types suggests that functional consequences of LGDs and missense variants may be similar and that loss-of function and haploinsufficiency may be the common pathomechanism.

To experimentally validate this hypothesis, we performed several functional assays on 17 different missense variants located in various functional domains. We thereby aimed to assess functional effects and to establish whether differences in these may contribute to the extremely large clinical variability. Of note, these assays can only be considered as additional evidence, but are neither necessary nor sufficient to prove pathogenicity of specific variants alone. Based on our data, tested missense variants do not seem to impair interaction with the SCF complex, which is thought to be mediated by the F-Box of FBXO11 (9). Currently knowledge about amino acids critical for this interaction is lacking. However, we found that the majority of *FBXO11* variants resulted in abnormal localization of the protein, either through exclusion from the nucleus or by accumulation in cytoplasmic aggregates. As the FBXO11-containing SCF E3-ubiquitin ligase complex functions primarily in the nucleus (10), the exclusion of FBXO11 from the nucleus is likely to lead to a loss of an important nuclear function. Consistently, known target proteins of FBXO11 also include various transcriptional regulators known to be localized to the nucleus such as BCL-6 and BAHD1, for example (10,48). Furthermore, for another F-box protein, FBXW7, subcellular mislocalization and exclusion from nucleus has been suggested as the key functional impairment for different somatic variants implicated in various cancers (49). On the other hand, abnormal cytoplasmic aggregation of FBXO11 observed for 10 missense variants from the F-Box, CASH 1 and 2 and Zf-UBR domains is also likely to interfere with its original nuclear function. The aggregates could possibly represent overexpression artifacts from abnormal proteins that overwhelm the cellular degradation machinery. It is, however, notable that these aggregates were also present when transfecting lower amounts of plasmid, in another cell type, and were hardly found in cells overexpressing wildtype FBXO11, therefore suggesting that aggregation is related to the specific variants. As the exact function of FBXO11 in neurons is not well understood, the consequences of the observed aggregates are not clear, but an effect through partial mislocalization of FBXO11 in these cytoplasmic aggregates seems plausible. We therefore consider that these missense variants may render FBXO11 more prone to aggregation likely leaving the protein non-functional, which has also been observed in other NDDs (50). For one *FBXO11* mutant construct, carrying a variant affecting the Zf-UBR domain, aggregates possibly partially co-localized with components of the SCF complex in the cytoplasm. Whether these complex aggregates represent SCF complexes and whether these complexes may be functional remains elusive and rather unlikely, especially in light of the nuclear role of the SCF complex (10).

In addition, about half of the variants tested showed reduced expression levels, partially overlapping with variants showing abnormal localization. This was true for all variants affecting the NLS and the F-Box domain. Several, but not all of the missense variants in the CASH 1 and 2 domains also led to reduced FBXO11 expression levels. *In-silico* mutational modelling data predicted destabilizing effects for variants in the CASH domains due to either steric clashes or loss of stabilizing interactions. Our functional data suggest that this destabilization can be reflected by either reduced expression and/or aggregation likely due to misfolding of the CASH domain. Interestingly, somatic *FBXO11* missense variants found in various cancer cell lines are mostly affecting CASH domains, are also predicted to lead to a loss of FBXO11 function (10), but are unique from germline variants seen in NDDs. Together with our expression data it therefore seems plausible that these variants with reduced FBXO11 levels result from an unstable protein, which is subsequently degraded. This would suggest that these variants act as hypomorphs and likely lead to a partial loss of FBXO11. However, we cannot rule out the possibility that synthesis of the affected mutant protein is impaired. In general, for missense variants in haploinsufficient genes it has been shown that they are more likely to function through destabilizing protein structure leading to a loss-of function than to gain-of function or dominant negative effects (51). Furthermore, pathogenic missense variants are enriched for variants affecting protein stability compared with benign variants (52).

Overall, we found that most *FBXO11* missense variants affect either subcellular localization, protein expression levels or both. Only one missense variant [p.(P905R)] did not show functional effects in our localization and expression assays. Effects of this and other variants on distinct functions such as for example ubiquitination of FBXO11 target proteins were not tested here, but would be interesting to explore in future studies. This does not rule out an effect of this variant on FBXO11 function, and based on the variant location, *de novo* occurrence and absence in population databases we still consider this variant likely pathogenic. It furthermore highlights the challenges in characterizing various functionally diverse missense variants in one gene, both molecularly and functionally. Such diversity of functional effects of missense variants has already been shown for other NDDs, for example for *TCF4* variants causing Pitt-Hopkins syndrome (MIM #610 954) or variants in *UBE3A* causing Angelman syndrome (MIM #105 830). For *UBE3A*, which encodes another nuclear E3 ubiquitin ligase, missense variants have been shown to affect subcellular localization, protein stability and catalytic activity (53). For *TCF4*, which encodes a transcription factor, missense variants can affect DNA binding, dimerization, transactivation capabilities as well as protein stability and subcellular localization (50). For both disorders, the general disease pathomechanism is haploinsufficiency, and the diverse functional effects are all predicted to abrogate the protein’s original function.

Our data suggest that location of missense variants in the FBXO11 protein influences their functional effects. Variants in the NLS and the F-Box affect localization and expression, variants in the Zf-UBR domain and the C-terminal region tend to affect localization only, whereas the effect of variants in the CASH domains is more variable. Therefore, variant location does not seem to be the sole determining factor, and prediction of functional defects based on variant location alone is likely not possible. In general, the functional diversity is expected to lead to similar downstream loss-of-function effects. For variants with altered localization only, specific gain-of function effects are not likely, but cannot be excluded. In line with this, levels of ID tend to be more severe in individuals with missense variants affecting subcellular localization only (0 out of 5 with mild DD/ID versus 4 out of 9 for other missense variants tested), but more cases would be necessary to see if this trend holds true. Clinical variability may therefore partially be explained not only by mutation type but also by its specific functional effects although to date numbers are still too small in different mutation subgroups to draw more detailed conclusions.

In summary, we expand the clinical and mutational spectrum of FBXO11-associated NDD and provide evidence that while some clinical variability may be influenced by mutation type and location, most missense variants and LGD variants are likely to function through a shared pathomechanism of loss-of function and haploinsufficiency.

## Materials and Methods

### Patients and patient material

Personal communication with colleagues following the initial report (2) and using GeneMatcher (54) and Decipher (55) allowed us to assemble the clinical and mutational data of 23 individuals with *FBXO11* variants. Testing in collaborating centers was performed either in the setting of routine diagnostics without the requirement for institutional ethics approval or within research settings approved by the ethical review board of the respective institutions (for details see Supplementary Material, Table S1). Variants were identified through either chromosomal microarray testing (I21 and I23), trio exome sequencing (I2–6, I9, I11–13, I16 and I18–20, I22), single exome (I8, I10 and I14), gene panel analysis (I1 and I17) or exome pool seq (I7). Paternity was confirmed for 15 cases through trio exome sequencing (also see Supplementary Material, Table S1). Informed consent for testing and for publication of mutational and clinical data was obtained from the individuals, their parents or legal guardians. All variants observed in affected individuals are annotated according to NM_001190274.1.

### *In-silico* analyses

Missense variants were tested for their potential predicted effects in splicing using SpliceAI (37). Alignments for conservation analysis of missense variants were performed using Clustalw2 (56,57). Identification of FBXO11 NLS was achieved using the online tool cNLS mapper (38). Structural modelling of the CASH domains was performed with HHpred (58) and Modeller (59) based on the crystal structures of the alginate epimerase AlgG [PDB:4OZZ, 4NK6; (60)]. The F-Box domain was modelled with Swiss-Model (61) based on the crystal structure of FBXL5 [PDB: 6VCD; (62)]. Mutations were modelled with Swiss-Model (61), and the Phe168 deletion was modelled with ModLoop (63). RasMol (64) was used for structure analysis and visualization.

### Plasmids construction

An expression plasmid containing human *FBXO11* (NM_025133.4) and the respective negative control plasmid were obtained from Sino Biologicals, Beijing, China (FBXO11-Myc: HG13948-CM and pCMV-Myc: CV014). Variants from affected individuals are annotated according to the longest *FBXO11* isoform (NM_001190274.1). Corresponding variants of both isoforms are indicated in Table 2.

Seventeen missense variants observed in affected individuals in this study or previously published (2) were introduced into the plasmid using a modified version of the Quik-Change site-directed mutagenesis kit (Stratagene, Agilent, Sanat Clara, CA, USA; Table 2). In addition, an expression plasmid containing the MEF2C open reading frame was used as a transfection and normalization control (MEF2C-FLAG: HG12320-CF, Sino Biologicals, Beijing, China).

### Immunofluorescence

HeLa cells were grown on coverslips, transiently transfected using jetPrime (Polyplus transfection, Illkirch, France) with either wildtype *FBXO11* or one of the mutant constructs and fixated with 4% paraformaldehyde in PBS for 10 min. Immunofluorescence stainings were performed three times. HeLa cells were transfected with 400 ng plasmid per 12 well. To confirm specificity of aggregates a test experiment was performed with 200 ng per 12 well in Hela cells as well as in HEK293 cells (500 ng per 12 well). Staining was performed with antibodies against FBXO11 (NB100-59826, Novus Biologicals, Centennial, CO, USA 1:200), Myc (M4439, Sigma-Aldrich, St. Louis, MO, USA 1:500), EEA1 (610 456, BD Biosciences, Franklin Lakes, NJ, USA 1:200), GOLGIN-97 (A-21270, Thermo Scientific, Waltham, MA, USA 1:100), LAMP-1 (sc-20 011, Santa Cruz Biotechnology, CA, USA 1:500), SEC31A (612 350, BD Biosciences, Franklin Lakes, NJ, USA 1:1000), SKP1 (2156, Cell Signaling, Cambridge, UK; Millipore, Burlington, MA, USA 1:50), CUL1 (sc-17 775, Santa Cruz Biotechnology, CA, USA 1:50), FK2 (04–263, Millipore, Burlington, MA, USA 1:500) at indicated dilutions. Secondary antibodies used were Alexa Fluor 488 goat anti-mouse (A11001), Alexa Fluor 546 donkey anti-rabbit antibodies (A10040), Alexa Fluor 488 goat anti-rabbit (A11008) and Alexa Fluor 546 donkey anti-mouse (A10036, all Thermo Scientific, Waltham, MA, USA). Nuclei were counterstained with DAPI (Serva, Heidelberg, Germany). Images were taken with a Zeiss Axio Imager Z2 Apotome microscope with a 63x objective and analyzed in ImageJ [v1.52; (65)].

### Protein expression analysis

HEK293 cells were transiently transfected with wildtype or mutant *FBXO11* and a plasmid containing *MEF2C*-FLAG as a control for transfection efficiency (1.2 μg of *FBXO11* plasmid and 500 ng of *MEF2C* plasmid per 6 well). Forty-eight hours post transfection cells were harvested in lysis buffer (100 mm TRIS–HCl pH 8, 150 mm NaCl, 1 mm EDTA and 1% Triton X-100). For western blotting, proteins were separated on stain-free 4–20% Mini-PROTEAN TGX Precast Protein Gels (Bio-Rad), and blots were stained with anti-Myc (1:5000), anti-FBXO11 (1:2500), anti-FLAG (7425, Sigma-Aldrich, St. Louis, MO, USA 1:5000) and anti-GAPDH (2118, Cell Signaling Technology, Cambridge, UK 1:5000) antibodies. Blots were stained with SuperSignal West Femto Maximum Sensitivity Substrate, scanned using the ChemiDoc Imaging System (Bio-Rad, 17 001 401) and analyzed using the Image Lab software version 6.0.0 (Bio-Rad). Band intensity of the target protein FBXO11-Myc was normalized to the expression of the transfection control MEF2C-FLAG. Significance was calculated using a one sample *t*-test with theoretical value set to 100.

### Co-immunoprecipitation

HEK293 cells were transiently transfected with wildtype or mutant *FBXO11* plasmids (1.5 μg per 6 well). Cells were scraped from the culture dish in lysis buffer (100 mm TRIS–HCl pH 8, 150 mm NaCl, 1 mm EDTA, 1% Triton X-100). Immunoprecipitation was performed with Protein A Mag Sepharose bead suspension (GE Healthcare, Boston, MA, USA), incubated with the sample and anti-Myc antibody (M4439, Sigma-Aldrich, St. Louis, MO, USA) at 4°C overnight. Subsequently, beads were washed in lysis buffer, and samples were eluted with 1x Lämmli buffer. SDS-page and western blotting was performed as described previously.

## Supplementary Material

HMG-2021-D-00350_Supplementary_material_060721_ddab265

Table_S1_050721_ddab265

Table_S3_050721_ddab265
