# Supplementary material for: De novo missense variants in FBXO11 alter its protein expression and subcellular localization
Source: Hum Mol Genet. 2021 Sep 9;31(3):440–54. doi: 10.1093/hmg/ddab265 (PMC8825234; doi:10.1093/hmg/ddab265)
Supplement: HMG-2021-D-00350_Supplementary_material_060721_ddab265 [file hmg-2021-d-00350_supplementary_material_060721_ddab265.pdf]

Supplementary material

De novo missense variants in _FBXO11_ alter its protein expression and subcellular localization

Anne Gregor[1,2]*, Tanja Meerbrei[1], Thorsten Gerstner[3], Annick Toutain[4,5], Sally Ann Lynch[6], Karen Stals[7], Caroline Maxton[8], Johannes R. Lemke[9], John A. Bernat[10], Hannah M. Bombei[10], Nicola Foulds[11], David Hunt[11,12], Alma Kuechler[13], Jasmin Beygo[13], Petra Stöbe[14], Arjan Bouman[15], Maria Palomares-Bralo[16], Fernando Santos-Simarro[16], Sixto Garcia-Minaur[16], Marta Pacio-Miguez[16], Bernt Popp[9], Georgia Vasileiou[1], Moritz Hebebrand[1], André Reis[1], Sarah Schuhmann[1], Mandy Krumbiegel[1], Natasha J. Brown[17,18], Peter Sparber[19], Lyusya Melikyan[19], Liudmila Bessonova[19], Tatiana Cherevatova[19], Artem Sharkov[20], Natalia Shcherbakova[20,21], Tabib Dabir[22], Usha Kini[23], Eva M.C. Schwaibold[24], Tobias B. Haack[14], Marta Bertoli[25], Sabine Hoffjan[26], Ruth Falb[14], Marwan Shinawi[27], Heinrich Sticht[28], Christiane Zweier[1,2]

```
                      135  138       168       185       206
Homo sapiens          GHRAKRARVSGK VLKIFSYLL CVCKRFSEL EVFEYTRPM
Macaca mulatta        GHRAKRARVSGK VLKIFSYLL CVCKRFSEL EVFEYTRPM
Rattus norvegicus     GHRAKRARVSGK VLKIFSYLL CVCKRFSEL EVFEYTRPM
Mus musculus          GHRAKRARVSGK VLKIFSYLL CVCKRFSEL EVFEYTRPM
Gallus gallus         GHRAKRARVSGK VLKIFSYLL CVCKRFSEL EVFEYTRPM
Xenopus tropicalis    GHRAKRARVSGK VLKIFSYLL CVCKRFSEL EVFEYTRPM
Danio rerio           GHRAKRARVSGK VLKIFSYLL CVCKRFSEL EVFEYTRPM
                      ************ ********* ********* *********

                        421          502       549|550    578
Homo sapiens          DHAQGIYED    HHGQTGGIY NGNQGGVYI IQIRTNSCPI
Macaca mulatta        DHAQGIYED    HHGQTGGIY NGNQGGVYI IQIRTNSCPI
Rattus norvegicus     DHAQGIYED    HHGQTGGIY NGNQGGVYI IQIRTNSCPI
Mus musculus          DHAQGIYED    HHGQTGGIY NGNQGGVYI IQIRTNSCPI
Gallus gallus         DHAQGIYED    HHGQTGGIY NGNQGGVYI IQIRTNSCPI
Xenopus tropicalis    DHAQGIYED    HHGQTGGIY NGNQGGVYI IQIRTNSCPI
Danio rerio           DHAQGIYED    HHGQTGGIY NGNQGGVYI IQIRTNSCPI
                      *********    ********* ********* *********

                        650          679       709       910
Homo sapiens          YDNGHGVLE    KIRRNKIWG FDNAMAGVW THDTDTLYD
Macaca mulatta        YDNGHGVLE    KIRRNKIWG FDNAMAGVW THDTDTLYD
Rattus norvegicus     YDNGHGVLE    KIRRNKIWG FDNAMAGVW THDTDTLYD
Mus musculus          YDNGHGVLE    KIRRNKIWG FDNAMAGVW THDTDTLYD
Gallus gallus         YDNGHGVLE    KIRRNKIWG FDNAMAGVW THDTDTLYD
Xenopus tropicalis    YDNGHGVLE    KIRRNKIWG FDNAMAGVW THDTDTLYD
Danio rerio           YDNGHGVLE    KIRRNKIWG FDNAMAGVW THDTDTLYD
                      *********    ********* ********* *********
```

Figure S1: Conservation of novel missense variants across species. Multiple sequence alignment was made using Clustalw2 (1, 2), variant positions are highlighted in green. Reference sequences for

different species: homo sapiens: NP_001177203.1, Macaca mulatta: AFJ72146.1, Rattus norvegicus: NP_853662.1, Mus musculus: AAI28480.1, Gallus gallus: XP_015133606.1, Xenopus tropicalis: XP_012819332.1, Danio rerio: XP_005169611.1.

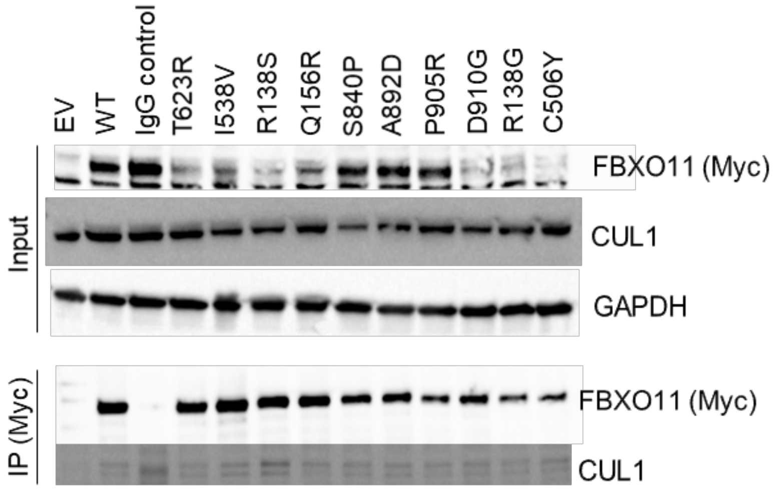

Figure S2: No impairment of SCF complex formation for FBXO11 variants. Co-immunoprecipitation experiments were performed with an anti-Myc antibody to precipitate wildtype and mutant FBXO11. CUL1 co-precipitates with wildtype and mutant FBXO11 suggesting that FBXO11 complex formation is not impaired.

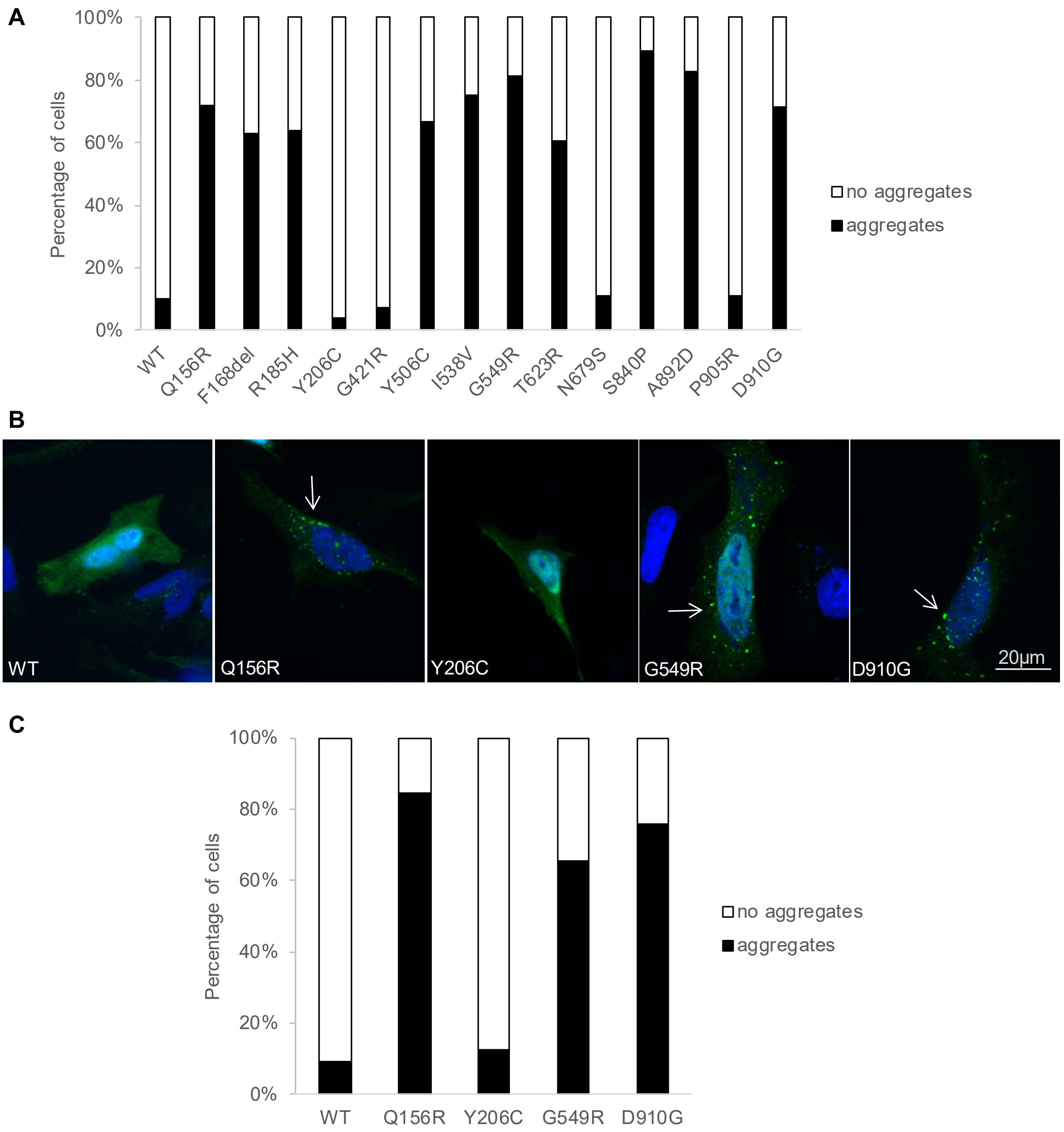

Figure S3: FBXO11 variants affect subcellular localization in HeLa cells. (A) Quantification of aggregate phenotype shown in Figure 3. At least 30 cells were analysed and scored for the presence or absence of aggregates for each condition. 400ng vector were transfected per 12 well for this experiment. (B) Representative images of several FBXO11 variants transiently transfected with 200ng vector per 12 well (half of the amount used in Figure 3). Cells were fixed 48h post transfection and stained with an anti-Myc antibody. Images were taken on a Leica DMI4000 fluorescence microscope. Scale bar 20µM. Arrows point to subcellular aggregates present in several mutants. (C) Quantification of aggregated

phenotype from (B). At least 20 cells were analysed and scored for the presence of absence of aggregates for each condition.

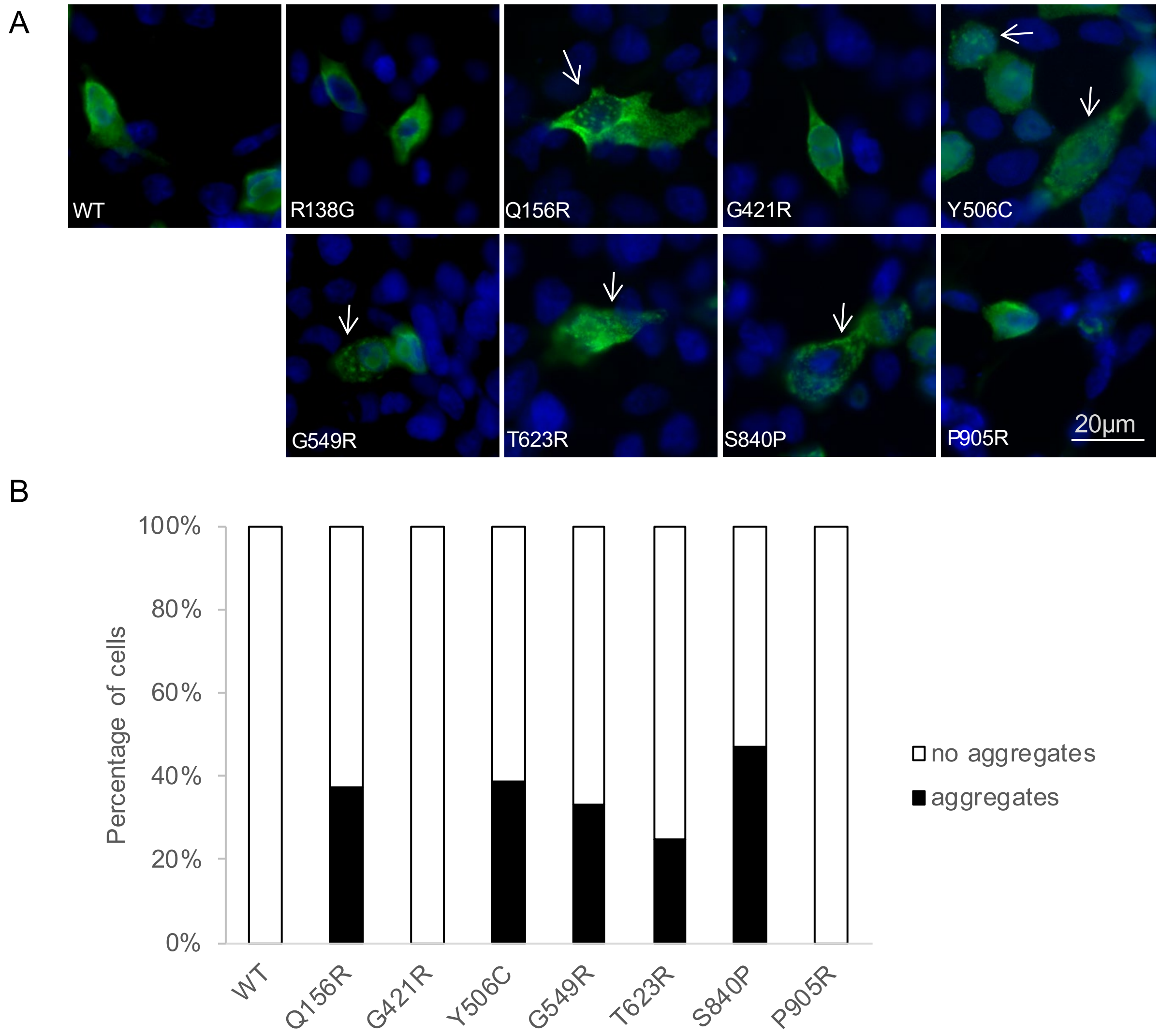

Figure S4: FBXO11 variants also affect subcellular localization in HEK293 cells. (A) Representative images of immunofluorescence on HEK293 cells transiently transfected with 500ng per 12 well of wildtype or mutant Myc-FBXO11. Mutant constructs affecting different domains and presenting with different phenotypes were selected. Cells were fixed 48h post transfection and stained with an anti-Myc antibody. Images were taken on a Leica DMI4000 fluorescence microscope. Scale bar 20µM. Arrows point to subcellular aggregates present in several mutants. (B) Quantification of aggregate phenotype. At least 20 cells were counted and scored for presence or absence of aggregates for each condition.

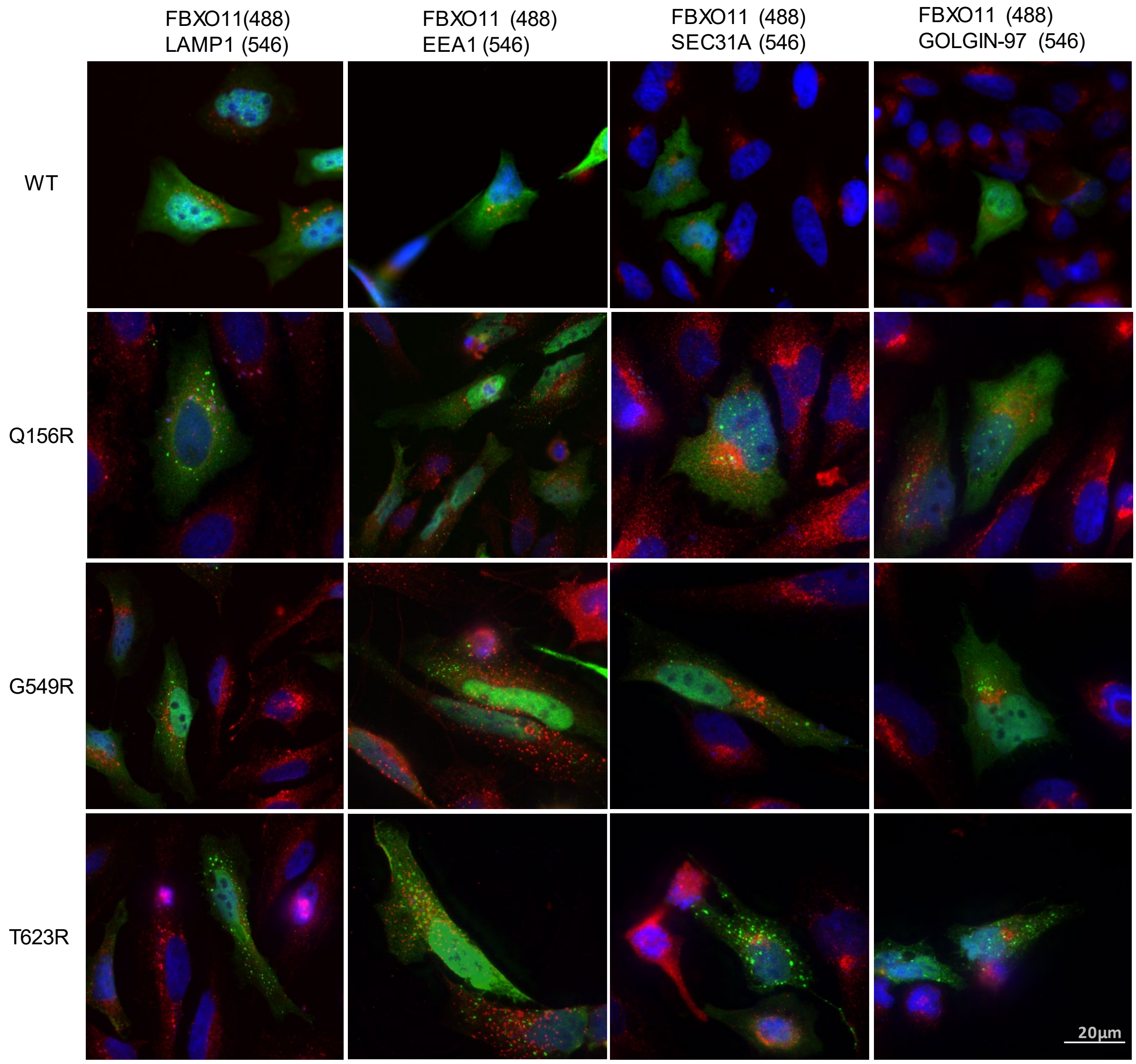

Figure S5: No co-localization of aggregates with subcellular organelles in HeLa cells. Immunofluorescence of FBXO11 (green) and subcellular organelle markers (red) for late endosomes (LAMP1), early endosomes (EEA1), Endoplasmic reticulum (SEC31A) and Golgi apparatus (GOLGIN-97) shows that mutant FBXO11 aggregates do not overlap with common subcellular organelles. Selected variants for different domains (G156R – F-Box domain, G549R – CASH 1, T623R – CASH 2) were tested in HeLa cells. Nuclei are stained with DAPI. Images were taken on a Axioimager Z2 with Apotome. Scale bar 20µM.

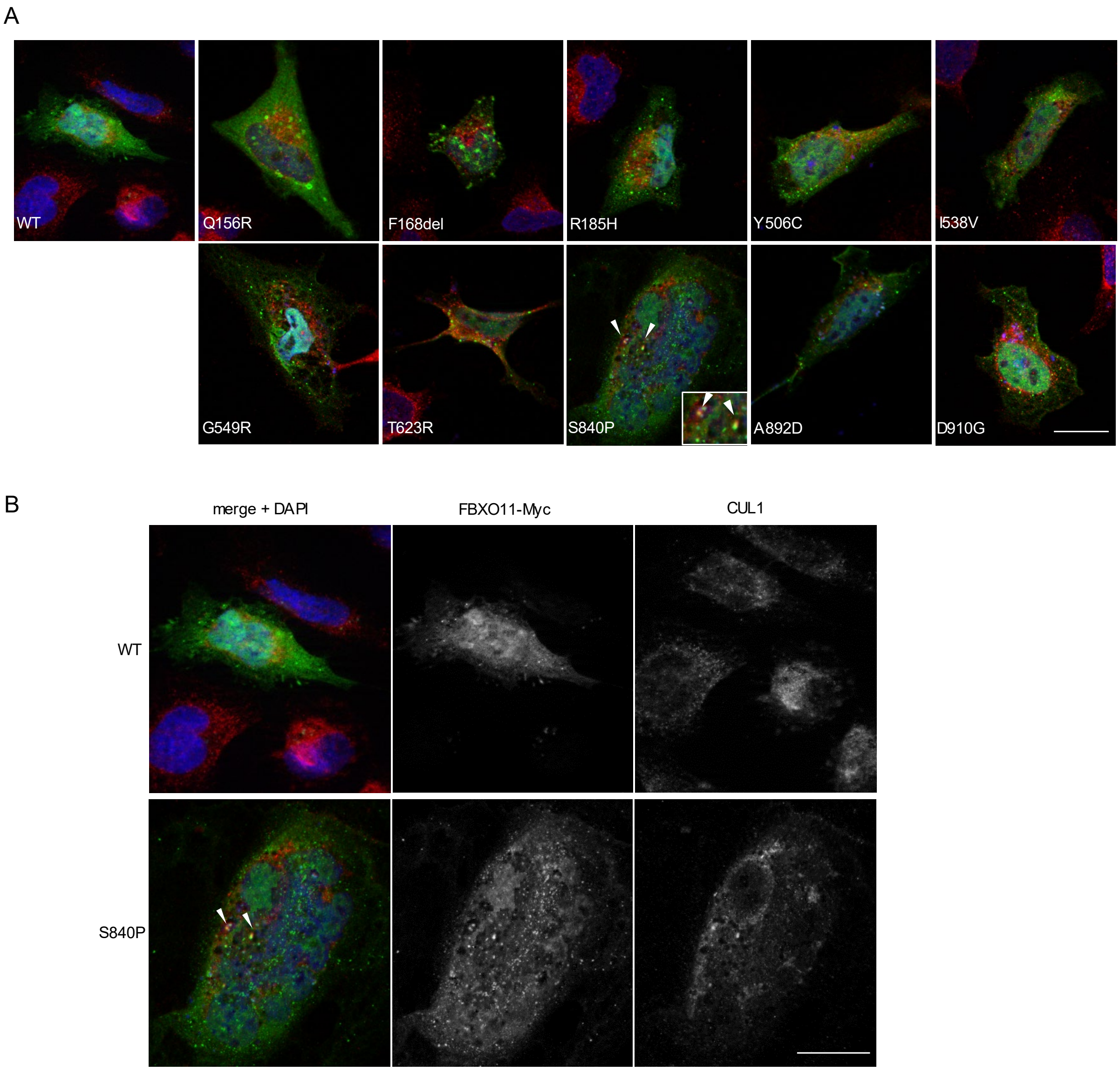

Figure S6: Rare co-localization of aggregates with SCF complex in HeLa cells. (A) Immunofluorescence staining of FBXO11 and CUL1, a component of the SCF complex, shows that aggregates of FBXO11 variant S840P partially co-localize with CUL1. All other variants show no co-localization. Variants that showed aggregate formation in initial FBXO11 stainings (Figure 3) were tested in HeLa cells. (B) Individual channels and merged image of WT FBXO11 and S840P variant highlighting partial co-localization with CUL1 in S840P variant. Nuclei are stained with DAPI. Images were taken on a Axioimager Z2 with Apotome. Scale bar 20µM.

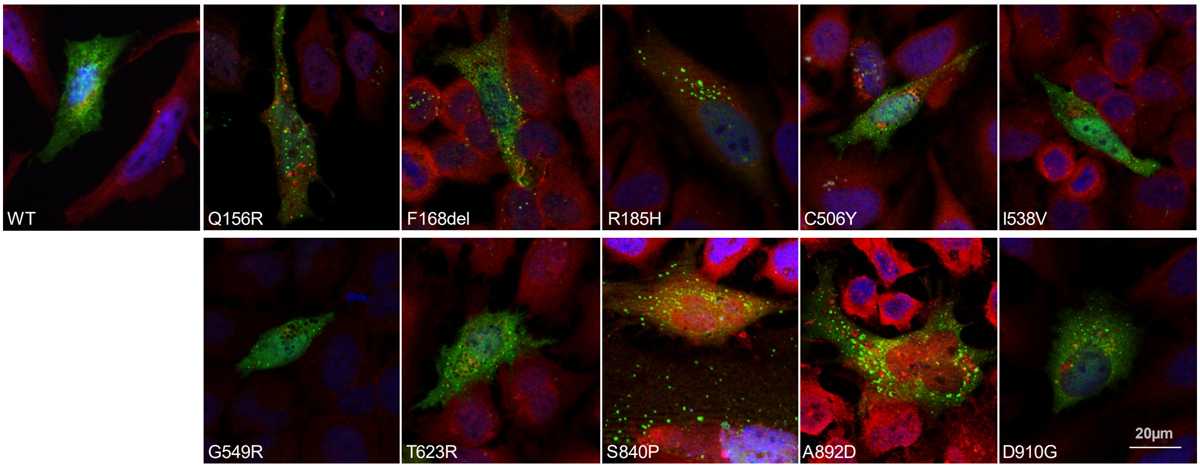

Figure S7: No co-localization of aggregates with ubiquitinated proteins in HeLa cells. Immunofluorescence staining of FBXO11 shows that mutant FBXO11 aggregates do not overlap with a marker for ubiquitinated proteins (FK2). Variants that showed aggregate formation in initial FBXO11 stainings (Figure 3) were tested in HeLa cells. Nuclei are stained with DAPI. Images were taken on a Axioimager Z2 with Apotome. Scale bar 20µM.

Table S1: Detailed clinical information on all 16 novel cases with de novo Fbxo11 aberrations.

See excel sheet.

Table S2: Results of various pathogenicity prediction programs for novel missense variants

**chromosomal position**	**variant**	**CADD**	**REVEL**	**Polyphen**	**Mutation Taster**	**SIFT**	**M-CAP**	**MPC**	**Condel**	**GERP**
hg19:chr2:g.48066596T>C	K135R	19.28	0.269	0.425 (B)	1 (D)	0.08 (T)	0.026 (PP)	0.313	0.18 (N)	5.09
hg19:chr2:g.48066588T>C	R138G	19.28	0.298	0.197 (B)	1 (D)	0.39 (T)	0.012 (B)	0.486	0.3 (N)	2.61
hg19:ch2:g.48063111T>C	R185H	32	0.624	0.999 (PrD)	1 (D)	0 (D)	0.119 (PP)	1.214	0.847 (D)	5.17
hg19:chr2:g.48066031C>T	Y206C	27.5	0.566	0.990 (PrD)	1 (D)	0 (D)	0.032 (PP)	0.919	0.816 (D)	5.5
hg19:chr2:g.48059625C>T	G421R	34	0.747	0.999 (PrD)	1 (D)	0 (D)	0.343 (PP)	1.29	0.919 (D)	5.95
hg19:chr2:g.48050394T>G	T502P	28.1	0.834	0.954 PrD)	1 (D)	0.01 (D)	0.168 (PP)	3.113	0.803 (D)	5.66
hg19:chr2:g.48049414C>T	G549R	32	0.882	0.998 (PrD)	1 (D)	0 (D)	0.125 (PP)	2.979	0.895 (D)	5.42
hg19:chr:g.48049411C>G	G550R	29.6	0.936	0.89 (PD)	1 (D)	0 (D)	0.370 (PP)	2.979	0.794 (D)	5.42
Hg19:chr2:g.48047565G>C	T578R	27.4	0.755	0.84 (PD)	1 (D)	0.01 (D)	0.180 (PP)	2.645	0.727 (D)	5.35
hg19:chr2:g.48045975T>G	H650P	26.4	0.771	0.959 (PrD)	1 (D)	0.04 (D)	0.222 (PP)	3.362	0.752 (D)	5.84
hg19:chr2:g.48040977T>C	N679S	24.4	0.723	0.998 (PrD)	1 (D)	0.13 (T)	0.196 (PP)	2.216	0.669 (D)	5.43
hg19:chr2:g.48040475T>C	M709V	23.5	0.711	0.714 (PD)	1 (D)	0.01 (D)	0.066 (PP)	2.09	0.496 (D)	5.43
hg19:chr2:g.48035312T>A	D910V	24.5	0.551	0.017 (B)	1 (D)	0 (D)	0.097 (PP)	3.229	0.28 (N)	5.72

Annotation based on NM_001190274.1, N: neutral, D: deleterious, B: benign, PD: possibly damaging, PrD: probably damaging, PP: possibly pathogenic, T: tolerated, variant highlighted in red indicate damaging/highly conserved variants, orange indicated potentially damaging variants

Table S3: Summary of clinical data of all novel and published cases with Fbxo11 aberrations.

See excel sheet.

Supplementary references

1 Goujon, M., McWilliam, H., Li, W., Valentin, F., Squizzato, S., Paern, J. and Lopez, R. (2010) A new bioinformatics analysis tools framework at EMBL-EBI. *Nucleic Acids Res.*, **38**, W695-699.

2 Sievers, F., Wilm, A., Dineen, D., Gibson, T.J., Karplus, K., Li, W., Lopez, R., McWilliam, H., Remmert, M., Soding, J. *et al.* (2011) Fast, scalable generation of high-quality protein multiple sequence alignments using Clustal Omega. *Mol. Syst. Biol.*, **7**, 539.
